# Silver Nanomaterials for Wound Dressing Applications

**DOI:** 10.3390/pharmaceutics12090821

**Published:** 2020-08-28

**Authors:** Priya Dharshini Krishnan, Dominik Banas, Ramya Devi Durai, Daniil Kabanov, Bozena Hosnedlova, Marta Kepinska, Carlos Fernandez, Branislav Ruttkay-Nedecky, Hoai Viet Nguyen, Awais Farid, Jiri Sochor, Vedha Hari B. Narayanan, Rene Kizek

**Affiliations:** 1 Department of Pharmacy, School of Chemical and Biotechnology, SASTRA Deemed University, Thanjavur 613-401, India; p.dharshi14@gmail.com (P.D.K.); ramya@scbt.sastra.edu (R.D.D.); 2 Department of Biochemistry, Faculty of Science, Masaryk University, Kamenice 753/5, 625 00 Brno-Bohunice, Czech Republic; dominik.banass@gmail.com (D.B.); dac100@mail.ru (D.K.); 3 Department of Research and Development, Prevention Medicals, Tovarni 342, 742 13 Studenka-Butovice, Czech Republic; brano.ruttkay@seznam.cz; 4 Department of Viticulture and Enology, Faculty of Horticulture, Mendel University in Brno, Valticka 337, 691 44 Lednice, Czech Republic; bozena.hosnedlova@post.cz (B.H.); jiri.sochor@mendelu.cz (J.S.); 5 Department of Biomedical and Environmental Analyses, Faculty of Pharmacy, Wroclaw Medical University, Borowska 211, 50-556 Wroclaw, Poland; marta.kepinska@umed.wroc.pl; 6 School of Pharmacy and Life Sciences, Robert Gordon University, Garthdee Road, Aberdeen AB10 7QB, UK; c.fernandez@rgu.ac.uk; 7 Department of Molecular Pharmacy, Faculty of Pharmacy, Masaryk University, Palackeho 1946/1, 612 00 Brno, Czech Republic; 8 Research Center for Environmental Monitoring and Modeling, University of Science, Vietnam National University, 334 Nguyen Trai Street, Hanoi 100000, Vietnam; nguyenviethoai@hus.edu.vn; 9 Division of Environment and Sustainability, Hong Kong University of Science and Technology, Room 4412, Clear Water Bay, Kowloon, Hong Kong, China; awais@ust.hk; 10 Department of Pharmacology and Toxicology, Faculty of Pharmacy, Masaryk University, Palackeho 1946/1, 612 00 Brno, Czech Republic

**Keywords:** nanosilver, therapeutic activity, antibacterial effect, synthesis route

## Abstract

Silver nanoparticles (AgNPs) have recently become very attractive for the scientific community due to their broad spectrum of applications in the biomedical field. The main advantages of AgNPs include a simple method of synthesis, a simple way to change their morphology and high surface area to volume ratio. Much research has been carried out over the years to evaluate their possible effectivity against microbial organisms. The most important factors which influence the effectivity of AgNPs against microorganisms are the method of their preparation and the type of application. When incorporated into fabric wound dressings and other textiles, AgNPs have shown significant antibacterial activity against both Gram-positive and Gram-negative bacteria and inhibited biofilm formation. In this review, the different routes of synthesizing AgNPs with controlled size and geometry including chemical, green, irradiation and thermal synthesis, as well as the different types of application of AgNPs for wound dressings such as membrane immobilization, topical application, preparation of nanofibers and hydrogels, and the mechanism behind their antimicrobial activity, have been discussed elaborately.

## 1. Introduction

**Figure 1:**
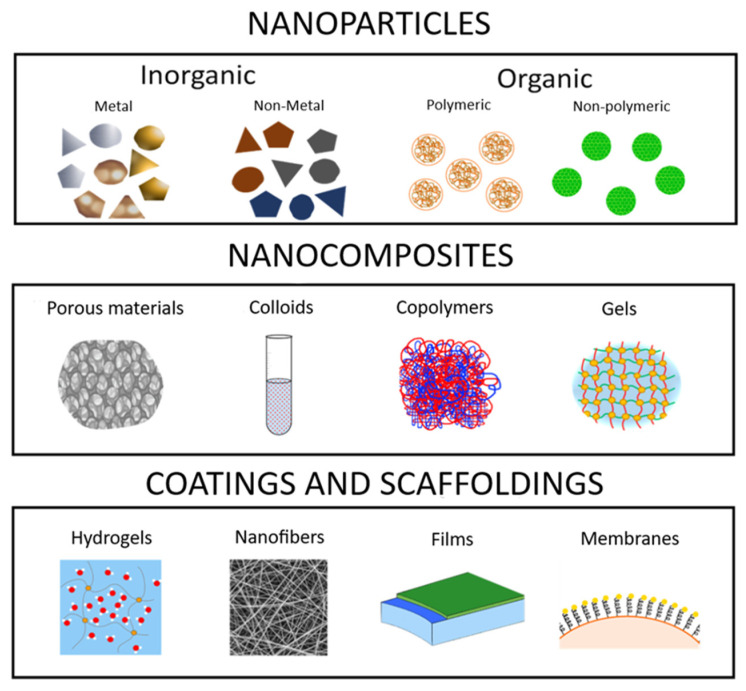
Nanomaterials with potential use for wound treatment. Nanomaterials cover several groups of materials including nanoparticles, nanocomposites and different coatings and scaffolding materials. Nanoparticles can be divided into two groups depending on their chemical basis, which comprises inorganic (metal—Ag, Au, Zn, Cu etc.; non-metal—for example, Se) and organic (polymeric and non-polymeric) nanoparticles. From the perspective of nanocomposites, four groups can be recognized: porous materials, colloids, copolymers and gels. For the wound coating and scaffolds, hydrogels, nanofibers, films and membranes can be used. Adapted from [25] under a CC BY 4.0 license.

Infected wounds represent a complex, non-trivial, problem which seriously jeopardizes the health and life of patients. One of the most frequent problems is a chronic ulcer with deep tissue damage in patients with diabetes, connected with abnormal fibroblast and keratinocyte proliferation, reduced cell migration and decreased angiogenesis, which can lead to impairment of vascularization, delayed wound contraction and subsequent formation of non-healing diabetic wounds [1]. Not only diabetic wounds but also leg ulcers, arterial insufficiency, pressure ulcers and burns impose substantial morbidity and mortality, deeply affecting quality of life, with high economic burden [2]. Generally, wounds can be divided into acute and chronic [3]. When chronic wounds were analyzed in a mouse model, a huge diversity of microorganisms formatting biofilm (for example, *Enterobacter cloacae*, *Streptococcus thermophilus*, *Propionibacterium acnes*) and without biofilm formation (*Achromobacter xylosoxidans*) were observed. Biofilms as sessile microbial consortia established in a three-dimensional structure are an important strategy implemented by microorganisms to survive in sometimes harsh environmental conditions [4]. Biofilms within the in vitro environment only are often referred to as immature when they have been growing for less than 24 h and mature when they have been growing for more than 24 h [5]. The ratio between different biofilm and non-biofilm creating bacterial types in a wound is dependent on type and time of treatment [6]. Thus, because a variety of different bacterial species live in the wounds, there is a widely acknowledged need for new antibacterial agents to address the global increase in resistance [7]. The level of resistance, especially against traditional antibiotics (for example, methicillin-resistant *Staphylococcus aureus* [8], penicillin-resistant *Enterococcus faecalis* [9] or multiresistant *Mycobacterium tuberculosis* [10]) is still increasing, and the rising incidence of antimicrobial resistance among pathogenic bacteria is one of the greatest healthcare challenges facing humanity today [11]. Thus, new approaches to overcome bacterial resistance are being tried, comprising by using plant extracts [12] or a combination of antibiotics and other antibacterial materials [13], especially nanomaterials, which comprise unary [14], binary (AgAu, AgPt NPs) [15,16] or multicomponent materials (CdZnSe-CdZnS nanoalloy) [17]. Nanotechnology is nowadays a flourishing scientific field, which associates nanoparticles with extraordinary functions and size-dependent physicochemical properties, differing significantly from the macroscopic forms of these elements. Nanomaterials comprise nanoparticles (NPs), which can be divided into inorganic and organic nanoparticles and nanocomposites, which can be grouped into the porous material, colloids, copolymers and gels. Finally, the applications of nanoparticles and nanocomposites in scaffolds and coatings are via hydrogels, nanofibers and films (Figure 1). Over the years, inorganic nanoparticles have been explored extensively in nanomedicine [18], electronics [19], drug delivery [20], biomedical devices [21], food sectors as package material [22,23], and dentistry such as: prosthetic, restoration, endodontic, orthodontic, periodontal and dental implant treatment. In addition, an improvement of mechanical properties of dental materials; inhibition of adhesion and bacteria growth of *Escherichia coli*, *Streptococcus mutans*, *Enterococcus faecalis*, *Pseudomonas aeruginosa*, *S. aureus* and the fungus *Candida albicanshave* been observed [24], etc.

**Figure 2:**
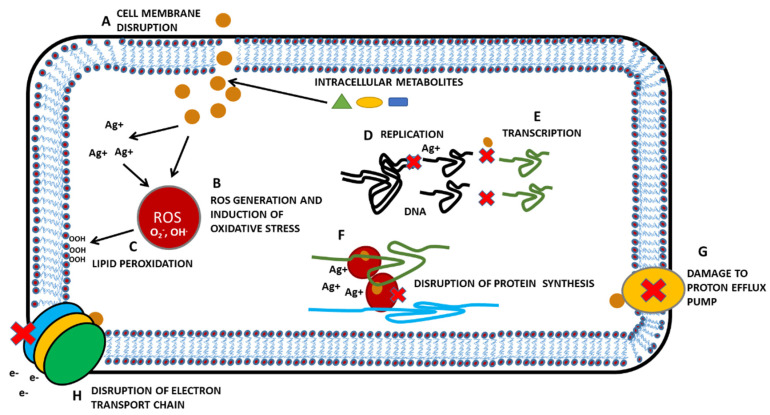
Effect of silver nanoparticles (AgNPs) on the single bacterial cell. (**A**) AgNPs penetrate through the cell membrane and cause its disruption; intracellular metabolites are released via the disrupted membrane. (**B**) AgNPs release silver ions (Ag^+^) to generate reactive oxygen species (ROS) and induce oxidative stress. (**C**) ROS are, for example, responsible for lipid peroxidation, which significantly changes the distribution of intracellular and extracellular metabolites and causes a change in membrane permeability, because lipid strains are repulsed. Interaction of AgNPs and Ag^+^ with proteins can affect (**D**) DNA enzymatic replication machinery, (**E**) DNA transcription, (**F**) translation of DNA to polypeptide/protein chain on ribosomes (red-brown ovals), (**G**) proton efflux pumps, (**H**) electron transport chain (represented by blue, yellow and green ovals) and creation of energetic sources of cells (ATP). Adapted from [31], Copyright Springer Nature, 2013.

Among the several inorganic NPs, silver NPs (AgNPs), due to their novel chemical, biological and physical properties as compared to their bulk form, have attracted the interest of researchers from the whole academic sphere. AgNPs have typical physical and chemical properties—for example, high thermal and electrical conductivity [26], surface-enhanced Raman scattering [27], chemical stability [28], catalytic activity [29] and non-linear optical behavior [30]. These properties take AgNPs to the top of the priority list, and they can be used in inks, in electronics and for medical purposes. Additionally, AgNPs are commonly known for their antibacterial, antifungal and antiviral properties. Mechanisms of effectivity comprise the disrupting of cell membranes, interaction with sulfhydryl groups of proteins, inactivation of respiratory enzymes, DNA modification, etc. (Figure 2). Microorganisms, however, have also mechanisms to adapt and to resist to metals (for example, reduced uptake, through efflux, chemical modification of metals, etc.) [31].

Thus, there is a need for a better understanding of these parameters on the efficacy and toxicity of AgNPs. Wide variety in size, concentration, shape, materials, polymers and surfaces of AgNPs are commonly being used [32].

**Figure 3:**
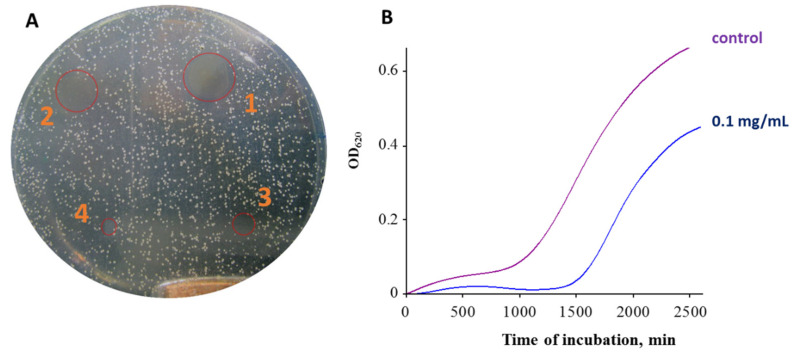
Two basic methods of screening of the antibacterial effect: (**A**) Effect of AgNPs in concentrations of 5 (1), 2.5 (2), 1.25 (3) and 0.625 mg/mL (4) in the agar plate disk diffusion test on *S. aureus* cells after 24 h of incubation. Growth inhibition zones are marked by red circles. (**B**) The growth curve of *S. aureus* without (control) or with 0.1 mg/mL AgNPs showed optical density of 620 nm (OD_620_), dependent on the time of growth.

The antimicrobial properties of AgNPs have been described and proven in various in vitro and in vivo studies, and their applications in food packaging, soaps, cosmetics, fabrics and wound dressing materials have also been reported [33]. There are a number of methods for antimicrobial susceptibility testing (AST) of bacteria, which can be divided into diffusion methods and dilution methods [34,35]. The most used diffusion methods are the agar disk-diffusion method and antimicrobial gradient method (Etest). The most used dilution methods are the broth dilution method and agar dilution method [36]. The broth dilution method can be further divided into the broth macrodilution method and broth microdilution method [36]. The broth macrodilution method has been standardized by the Clinical and Laboratory Standards Institute (CLSI) for testing bacteria that grow aerobically [37]. The broth microdilution method is standardized by CLSI for both anaerobic [38] and aerobic bacteria [39]. As a representative example of the agar plate disk diffusion test [40] and the broth microdilution test (a measurement of growth curves) [41], our own experimental results for green-synthesized nanoparticles are shown in Figure 3.

**Figure 4:**
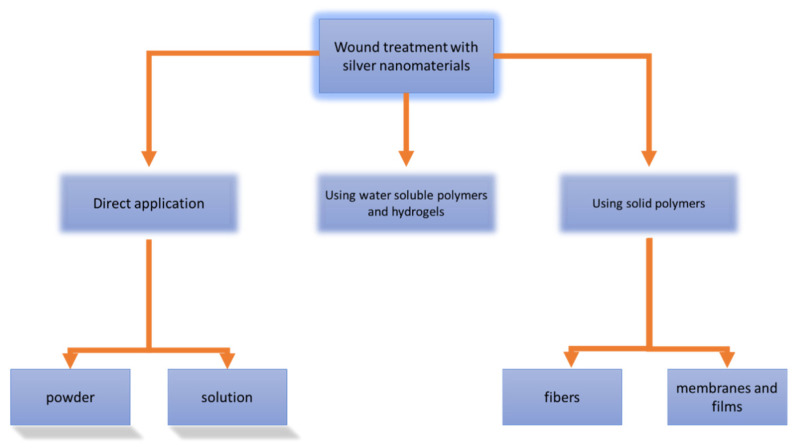
Wound treatment with silver nanomaterials. Nanomaterials can be applied directly to the wound (solution or powder nanoparticles) or nanomaterial-containing cloth and fabrics can be used. Hydrogels and water-soluble polymers are being used as non-compact materials (they are easily rubbed on the skin); on the contrary, compact materials comprising fibers, membranes and films are firm and non-soluble.

**Figure 5:**
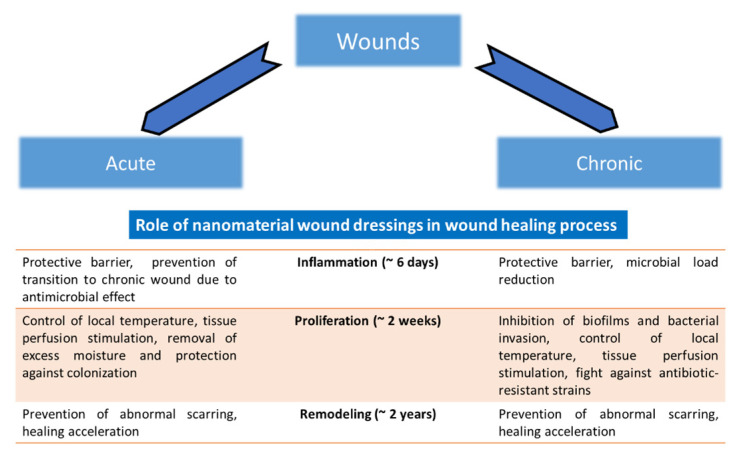
Summary of antimicrobial wound dressings with nanomaterials. The role of wound dressings using nanomaterials in various phases of wound healing (which comprise inflammation, proliferation and remodeling of the wound) is shown (the approximate duration of each phase is indicated in parentheses). Mostly, the effect is achieved due to the physical protection of the wound, acceleration of healing and broad antimicrobial action. Adapted from [25] under a CC BY 4.0 license.

AgNPs are also used in water filter membranes for point-of-care water disinfection, utilizing their antimicrobial properties [42]. The AgNPs can be used in all physical forms—for example, in colloidal form (in enamel coating and paints), liquid form (cosmetics) and solid form (blended with polymer scaffolds). For wounds, different types of treatment with silver and AgNPs were proposed and they are shown in Figure 4. Application of nanomaterials on the wound can be direct, in the form of powdered AgNPs or their solutions. The second possibility is the use of non-compact materials for wound treatment as water-soluble polymers and hydrogels. The third possibility is the use of solid polymers (fibers, membranes and films). The role of nanomaterial wound dressings in the wound healing process, in general, is shown in Figure 5. One problematic aspect of AgNPs wound dressings is that there are reported cases of bacterial resistance to AgNPs. For example, resistance to AgNPs attributed to *sil* genes has been reported in clinical isolates of *Klebsiella pneumoniae* and *Enterobacter cloacae* from burn cases [43]. Thus, the continuous development of a new combination of nanomaterials, antibiotics, polymers, methods of incorporation and types of reduction of silver to obtain various shapes of NPs is essentially necessary.

## 2. Methodology of the Review

**Figure 6:**
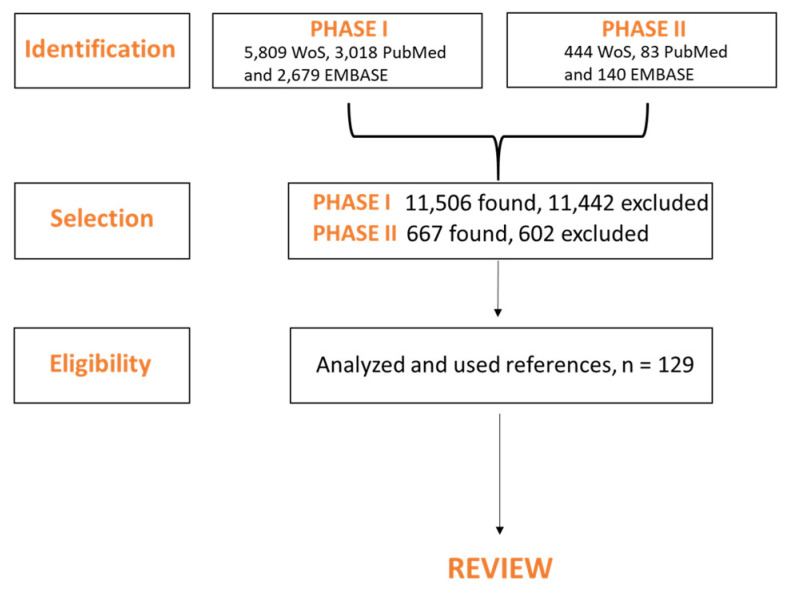
Flowchart of choice of articles for review, “Silver Nanoparticles for Wound Dressing Applications“. In the first phase, articles were searched according to the following keywords: “silver nanoparticles in wounds”; “silver nanoparticles and therapeutic application”; “chemical synthesis of AgNPs”; “green synthesis of AgNPs”; “irradiation synthesis of AgNPs”; “thermal synthesis of AgNPs”. The following databases were used: Web of Science (WoS) core collection; PubMed and Embase, searching since 2000. Completely, more than 11,000 articles were found (5809 WoS, 3018 PubMed and 2679 Embase). Condensate extraction of articles from this phase was used for writing introduction and parts about the synthesis of particles. In the second phase, we were interested only in articles with the keywords “AgNPs for wound dressing applications”. More than 600 articles were found (444 WoS, 83 PubMed and 140 Embase). Some of these articles were used for writing the main section concerning different means of preparing AgNPs wound dressings. It is seen that using AgNPs, especially in wound dressings, comprises the effort of many work teams from around the world; thus, it is impossible to describe all techniques, materials and types of synthesis of AgNPs. We tried to choose several representative examples for all types of synthesis and the most frequent using polymeric materials to give the reader a complex view of the problematic nature of using AgNPs in wound dressing. The articles which were preferred were articles from 2007, which describe mainly the use of AgNPs. The articles were selected also upon impact factor (average impact factor is 6.31) and quartile of the journal (Q1 and Q2). Finally, 129 articles were chosen to write this review.

The methodology of this review is summarized in Figure 6.

This paper aims to summarize the various methods adopted for the synthesis of AgNPs that include chemical, green, irradiation and thermal synthesis and routes of application of these nanoparticles through membrane immobilization, topical application, nanofibers and hydrogel system for wound dressing (see Figure 1). In this review paper, every aspect of AgNPs, from synthesis to their utilization in wound dressing applications against various bacterial and fungal strains, has been discussed.

## 3. Synthesis of AgNPs

**Figure 7:**
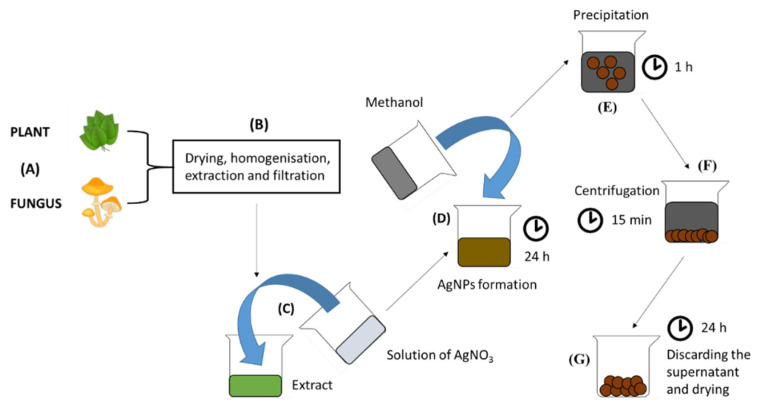
The process of preparation of AgNPs. (**A**) A plant/fungus is (**B**) homogenized, extracted, centrifugated and filtrated; subsequently (**C**), silver ions in the form of 0.1 M AgNO_3_ are added in a 1:1 (*v*/*v*) ratio. (**D**) AgNPs are formed within 24 h. (**E**) AgNPs are precipitated with methanol in a 1:1 (*v*/*v*) ratio for 1 h. (**F**) The solution of AgNPs is centrifuged for 15 min, 4000 *g*. (**G**)The supernatant is discarded and AgNPs are dried by lyophilization for 24 h.

This review aims to conclude the newest findings of the antibacterial effect of AgNPs connected with covering materials for wound dressing and healing. As mentioned above, four types of AgNPs synthesis are described, namely chemical, green, irradiation and thermal. In this section, the basic characteristics of each type of synthesis are briefly described. In chemical synthesis, the Turkevich method and Brust–Schiffrin synthesis are mainly used [43]. The Turkevich method is based on the reduction of the boiling solution of silver salt with a solution of citrate salt. The Brust–Schiffrin method is mainly used for golden nanoparticles, but applications for other metals were also described [44]. Stabilization of AgNPs synthesis is ensured by using surfactants (thiols, amines, acids and alcohols) or polymers (poly(vinyl alcohol), poly(vinylpyrrolidone), poly(ethylene glycol), poly(methacrylic acid) and polymethyl methacrylate) [45]. Green synthesis represents a low-cost, environmentally friendly way of producing AgNPs without using high temperatures and pressures [46]. Typically, the plant [46,47,48,49,50], fungus [51,52,53] or bacteria extract is mixed with silver ions containing salt (silver nitrate is mainly used [46,47,48,49,50,51,52,53]) and bioactive molecules of extracts reduce silver ions to elementary silver. A huge advantage of using plant extracts is the fact that many plants contain health-beneficial compounds, also known from their use in conventional medicine (for example, paclitaxel, vincristine or artemisinin) [54]. After nucleation, the AgNPs can be precipitated with alcohol [55] (for example, methanol, ethanol or isopropyl alcohol). Model synthesis is shown in Figure 7.

**Figure 8:**
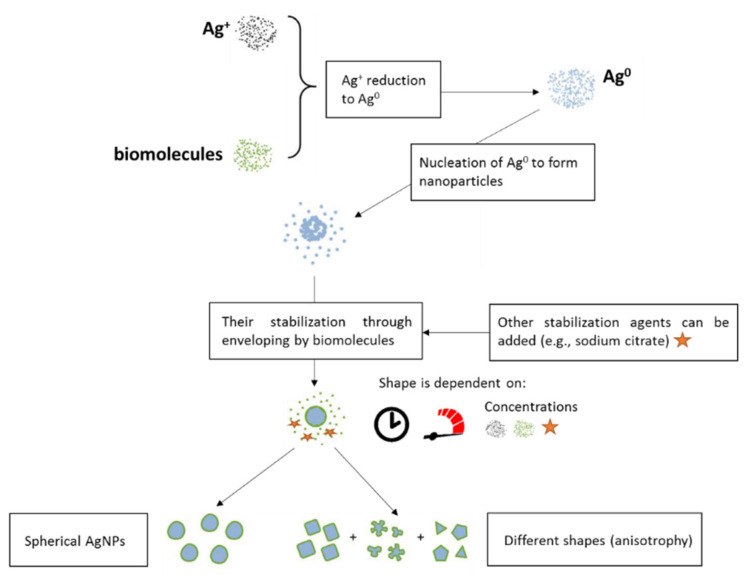
A model example of green synthesis of AgNPs. As mentioned in Figure 7, the green synthesis of AgNPs is based on using extracts from plants or fungi, which contain a giant spectrum of different oxidative and reductive agents. These molecules reduce silver ions (Ag^+^) to elementary silver (Ag^0^). Compounds which influence the reduction and capping of originating AgNPs are, for example, proteins, alkaloids or polysaccharides. Different types of nanoparticles can be prepared depending on reaction conditions (metal concentration, extract concentration, length of synthesis, stabilization agents, etc.).

The process of reduction of Ag^+^ to Ag^0^ with biomolecules relates to the high reduction power of the biomolecule cocktail. Depending on the extract composition, the velocity of stirring and time of nucleation of AgNPs, AgNPs with different shapes can be obtained (Figure 8).

Irradiation synthesis [56,57,58] is connected with radiation of precursors or intermediate products of reaction with electromagnetic radiation with different wavelengths. Only specialized laboratories with a suitable radiation source and in respect to the difficult legislature can achieve this type of synthesis. Jia et al. [56] prepared gold nanoclusters using triple helix glucan Lentiman via microwave-assisted synthesis and subsequently employed them as seeds for Ag-Au nanoparticle alloy; these nanoparticles increase in size with higher Ag amounts [56]. Mixed cellulose nanofibrils and AgNPs composites were prepared by UV radiation [57]. Graphene oxide (GO)-supported AgNPs nanocomposite was prepared via polyvinyl-pyrrolidone (PVP) and isopropanol (IPA)-assisted gamma irradiation [58].

Thermal synthesis is based on a thermal reduction of silver salt. A representative example is the thermal reduction of AgNO_3_ [59] in an environment of poly(*N*-isopropylacryamide-*co*-2-acrylamido-2-methylpropane sulfonic acid) (NIPAMSA) to form the hybrid hydrogels with incorporated AgNPs.

## 4. AgNPs Immobilization into Membrane and Composite Material

Membrane and composite material-immobilized nanoparticles can have a plethora of different functions (e.g., disinfection, catalytic activity). An example which can be mentioned as a composite material created by Liang et al. is a material based on a pre-treated egg-shell membrane with procyanidin to reduce silver ions into nanoparticles incorporated into the membrane structure. This membrane had catalytic activity consisting of the conversion of 4-nitrophenol to 4-aminophenol [60]. Silver ions reduced with ascorbic acid formed nanoparticles incorporated into polyethersulfone membranes and showed antibacterial activity against *S. aureus*, *S. albus* and *E. coli* when tested on bacterial plates [61]. Hanif et al. [62] used a non-toxic and environmentally friendly method based on tannic acid-mediated silver salt layer-by-layer in-situ reductions. This method was used for the preparation of a cellulose-AgNPs composite with uniform and controlled size and distribution of AgNPs for point-of-use water disinfection, which was tested successfully on *E. coli* bacteria. In the study published by Dong et al. [63], casein-coated AgNPs were embedded into acetate-cellulose membrane for control of biofouling. AgNPs effectively suppressed the growth of *Serratia marcescens*, and specifically, membranes with AgNPs displayed a decrease in microbial growth by 59–99% after this concentration was used [63]. Silver nanoparticles incorporated into the reverse osmosis membrane with a diameter of approximately 30 nm significantly reduced (by 64.6%) *Pseudomonas* sp. biofilm formation after 14 days of continuous cultivation [64]. Another study which was proposed by Saraswathi et al. [65] was based on polydopamine-coated poly(ether imide) membranes with incorporated AgNPs. Membranes showed antibacterial activity against both Gram-negative and Gram-positive bacteria and facilitated the separation of toxic contaminants. In general, it can be concluded that AgNPs can be used in drinking water treatment and as a pseudo-enzymatic catalyst in the reaction. Additionally, AgNPs-incorporated membranes are used for wound treatment; representative examples are shown in Table 1.

### 4.1. Chemical-Synthesized AgNPs Incorporated into Membranes and Composite Materials

The bacterial cellulose (BC) membrane has been utilized as a template for the in-situ preparation of AgNPs through the chemical reduction method. The lyophilized cellulose membrane with AgNPs was placed on the *E. coli* or *S. aureus* grown on an agar plate and incubated at 37 °C for 24 h. The sample derived from maltose with a silver content of 1.06% realized >99.99% killing ratio in the number of viable *E. coli,* when cultivated on maltose after a contact period of 18 h. Therefore, 1.2 mg of the silver-containing composite was sufficient to ensure no detectable growth of *E. coli* in 0.1 mL of medium with a bacteria concentration of around 108 CFU (colony forming units)/mL [66].

Levi-Polyachenko et al. [67] prepared chitosan membrane by the solvent casting method and loaded it with a different concentration of hexagonal AgNPs synthesized through the chemical route to demonstrate the synergistic wound healing activity. The developed hexagonal AgNPs represented a new addition to the chitosan wound dressing materials, as they could facilitate cell proliferation, mitigate bacterial infection and generate mild hyperthermia for the delivery of small drug molecules [67].

Another chemical method for the synthesis of AgNPs using AgNO_3_ as a precursor was reported. The generated nanoparticles were loaded into casted poly(vinylpyrrolidone)-chitosan (PVP-chitosan) membrane. The PVP-chitosan-silver composite film found higher antibacterial activity than both chitosan and AgNO_3_ alone. The L929 cell lines were used for a cytotoxicity study and adult male albino rats (140–180 g) were selected for the in vivo wound healing study. The prepared film exhibited higher wound healing efficiency than the cotton gauge, 100% chitosan and other reported chitosan-based dressings [68].

The chemical reduction of AgNO_3_ resulted in the formation of AgNPs, which were loaded into the chitin hydrogel. These hydrogels were lyophilized to obtain the AgNPs-impregnated chitin membrane. These chitin-nanosilver composite scaffolds exhibited good blood clotting ability and were also found to be bactericidal against *S. aureus* and *E. coli* [69].

Maneerung et al. isolated BC [70] produced by *Acetobacter xylinum*. It was used as a template for the synthesis of AgNPs through the chemical method. The freeze-dried silver nanoparticle-impregnated BC exhibited strong antimicrobial activity against *E. coli* (Gram-negative) and *S. aureus* (Gram-positive) bacteria [70].

### 4.2. Green-Synthesized AgNPs Incorporated into Membranes and Composite Materials

A composite sponge was made using konjac glucomannan (KGM) as the polymer and loaded with AgNPs that were prepared through a green synthesis method using egg white as a reducing agent. Animal models showed that the KGM-AgNPs composite sponges had effectively accelerated the wound healing process. The histological findings showed that they promoted fibroblast growth and accelerated epithelialization [71].

The regenerated cellulose and chitosan were blended and made into a solution for film casting in the presence of AgNO_3_ as a precursor for AgNPs production through the green reduction method. The tea grains (*Camellia sinensis*) were used as a reducing as well as a capping agent for the NPs. Evaluation of the optimized composite film for temporary biological wound dressing materials on the experimental wounds of rats had revealed the significant wound healing of the experimentally treated wounds faster than the control wounds [72].

The solvent casting method was performed for the preparation of chitosan-polyvinyl alcohol (PVA)-curcumin (CU) composite membrane loaded with AgNPs. The AgNO_3_ solution precursor was added to the composite mixture, which led to the formation of AgNPs through the reduction of the precursor by chitosan and PVA. The antimicrobial and antifungal activity of the chitosan-PVA-AgNPs films demonstrated significant effects against *E. coli, Pseudomonas, Staphylococcus, Micrococcus, C. albicans* and *P. aeruginosa*. To improve the therapeutic efficiency further, CU was encapsulated along with chitosan-PVA-AgNPs nanocomposite films, which showed enormous growth inhibition of *E. coli* compared to plain CU and chitosan-PVA-AgNPs film alone [73].

The BC produced by *Acetobacter xylinum* was isolated and dipped in AgNO_3_ and AgCl solution to produce AgNPs. In-situ production of NPs was performed using the membrane as a template. The AgCl NPs-impregnated membrane exhibited high hydrophilic ability and strong antimicrobial activity against *E. coli* and *S. aureus* [74].

### 4.3. Irradiation-Synthesized AgNPs Incorporated into Membranes and Composite Materials

Chitin membranes loaded with AgNPs were prepared by the solvent casting method in the presence of 5% LiCl and dimethylacetamide (DMA) solvent system. The NPs were prepared by the gamma irradiation method, using AgNO_3_ as the precursor. The chitin membranes were loaded with ascending concentrations of AgNPs from 30 to 100 ppm. The chitin membranes with 100 ppm AgNPs showed promising antimicrobial activity against common wound pathogens (*P. aeruginosa* and *S. aureus*) [75].

### 4.4. Thermal-Synthesized AgNPs Incorporated in Membranes and Composite Materials

In-situ preparation of AgNPs inside the BC membrane by thermal reduction at 80 °C was executed to obtain AgNPs-loaded BC scaffold. The results showed that AgNPs-BC exhibited significant antibacterial activity against *S. aureus*. Moreover, AgNPs-BC allowed attachment and growth of rat fibroblasts with low cytotoxicity. The fresh epidermal and dermis thicknesses of the skin treated with AgNPs-BC samples were 111 and 855 μm, respectively, which was significantly higher than 74 and 619 μm for BC-treated groups and 57 and 473 μm for untreated control wounds [76].

## 5. Powdered AgNPs and Topical Application

Powdered AgNPs are used for incorporation into different types of clothing and dressings. Representative examples are summarized in Table 2.

### 5.1. Chemical-Synthesized Powdered AgNPs and Topical Application

The powdered AgNPs were successfully prepared via the addition of AgNO_3_ to alkali dissolved starch followed by precipitation with ethanol. AgNPs aqueous suspensions were prepared from powder AgNPs by dispersion and dilution with water. The cotton fabrics were impregnated with AgNPs by dipping in the solution with various concentrations. The results of potent healing using fabrics treated with 250 ppm AgNPs were similar to the Dermazin cream. Moreover, the anti-inflammatory effect of AgNPs was nearly equivalent to a 20-mL dose of the reference indomethacin drug [77].

The wound dressing materials coated with chemically synthesized AgNPs were tested for their antibacterial activity. Rapid healing and improved cosmetic appearance were observed in a dose-dependent manner. Furthermore, through quantitative PCR, immunohistochemistry and proteomic studies, the positive effects exerted by AgNPs by their antimicrobial properties, reduction in wound inflammation and modulation of fibrogenic cytokines were demonstrated [78].

### 5.2. Green-Synthesized Powdered AgNPs and Topical Application

Duran et al. synthesized AgNPs using *Fusarium oxysporum*. AgNPs were loaded inside the clothes and tested for their antibacterial activity against *S. aureus*. AgNPs demonstrated a 99% reduction in bacterial counts [79].

Sundaramoorthi et al. prepared AgNPs from *Aspergillus niger* using AgNO_3_ as a precursor. AgNPs incorporated into wound dressings had antibacterial activity and formed inhibition zones: 15 mm for *S. aureus*, 11 mm for *B. subtilis*, 10 mm for *E. coli* and 14 mm for *P. aeruginosa* [80].

## 6. Nanofibers

Nanofibers as materials can be used as a platform in Raman spectroscopy [81], in immunoanalysis [82], for air filtration [83] and as a pseudo-enzyme [84]. As can be seen, the AgNPs, besides their important wound treatment activity, which is summarized in Table 3 below, can be used also for disinfection and catalytic purposes.

### 6.1. Chemical Synthesis of AgNP-Containing Nanofibers

The chemically synthesized AgNPs were added to the electrospinning solution containing collagen to obtain the nanofibers. The in-vivo study proved that the rate of wound healing of the composite nanofiber mats was accelerated compared with plain collagen nanofibers. Histology analysis revealed accelerated re-epithelization, collagen production and better wound contraction with AgNPs composite collagen nanofibers [85].

The PVP nanofibers loaded with AgNPs that were reduced from precursor by *N*,*N*-dimethylformamide (DMF) were reported. The PVP-containing AgNPs could be used to introduce the nanoparticles into any other polymer nanofibers that are miscible with PVP. The PVA nanofiber mats were strong enough to act as antimicrobial separation filters [86].

An electrospun fiber made of poly(methyl methacrylate-*co*-dopamine methacrylamide) (PMMDM) was dipped into the AgNO_3_ solution for on-surface synthesis of AgNPs. The PMMDM-AgNP composite nanofibers containing 1% NPs achieved desirable antibacterial activity against both Gram-negative and Gram-positive bacteria, while not significantly affecting the viability of mammalian cells. The product was also observed with a rapid release of the AgNPs within 24 h, followed by a sustained release for 5 days thereafter [87].

The collagen fibrils were isolated from the tendons of Wistar rats and allowed to crosslink with plumbagin (PBG)-caged AgNPs that were prepared using the chemical oxido-reduction method, using PBG and AgNO_3_ as precursors. Crosslinking of collagen with PBG resulted in a uniform alignment of collagen fibrils to form orderly aligned porous structured scaffolds with potent antibacterial activity and enhanced ability to promote cell proliferation and wound healing [88].

The gelatin nanofibers prepared by the electrospinning technique were incorporated with AgNPs that were prepared in-situ by the chemical reduction method. The fibers were further crosslinked with glutaraldehyde to enhance mechanical stability. The antibacterial activity of the fibers was greatest against *P. aeruginosa*, *S. aureus*, *E. coli* and methicillin-resistant *S. aureus* [89].

The electrospun nanofibers of poly(ethylene oxide)-poly(caprolactone) (PEOPCL) blend were prepared in dimethylformamide solvent. The precursor AgNO_3_ was added to the electrospinning solution, which was chemically reduced by DMF and poly(ethylene oxide) (PEO). In addition, the PEO acted as a stabilizer for the formation of NPs. The composite nanofibers possessed antibacterial potential against antibiotic-resistant *E. coli* [90].

A wet spinning technique was carried out in the CaCl_2_ precipitation bath for the preparation of alginate fibers. The chemically reduced AgNPs solution was mixed with a spinning solution, which resulted in AgNPs-incorporated alginate fibers. AgNPs-loaded fibers could be easily applied in a wound healing paradigm, which could promote the repair process through the promotion of fibroblast migration to the wound area, reduction of the inflammatory phase and an increase in epidermal thickness in the repaired wound area, thereby improving the overall quality and speed of healing [91].

### 6.2. Green Synthesis of AgNPs-Containing Nanofibers

A green method for the synthesis of AgNPs was reported through the electrospinning technique in the presence of chitosan as a reducing agent along with PVA to obtain nanofibers. The results showed the superior qualities of the fibers and synergistic antibacterial effects by the combination of chitosan with AgNPs [92].

Very recently, El-Aassar et al. published a methodology to obtain silver-containing nanofibers by using polygalacturonic and hyaluronic acid. Successful AgNPs synthesis by PGA was verified by UV-vis spectral maxima ranging between 410 and 415 nm and a transmission electron microscopy image of 8.6 nm average particulate diameter. AgNPs components of (Ag-PGA/HA)-PVA nanofiber exhibited robust zone inhibition antibacterial activity against both Gram-positive and Gram-negative bacteria [93].

Another green synthesis method was also reported for developing AgNPs using *P. nigrum* leaf extract. The NPs solution was mixed with polycaprolactone (PCL) solution during electrospinning to obtain the nanofibers. The fabricated material showed excellent antibacterial activity against both *S. aureus* and *E. coli,* which also confirmed the ability to prevent bacterial colonization in wounds covered with this composed material [94].

### 6.3. Irradiation Synthesis of Silver Nanofibers

Nanofibers of silk fibroin were prepared using an electrospinning technique and loaded with AgNPs synthesized from the AgNO_3_ precursor using the gamma irradiation method. AgNPs-coated nanofibers effectively inhibited the growth of *S. aureus* and *P. aeruginosa* when the coating solution containing colloidal AgNPs was 4 mM or equivalent to 50.4 ng/cm^2^ of adsorbed AgNPs [95].

## 7. AgNPs-Hydrogels

AgNPs incorporated in poly(*N*-iospropylacryamide-*co*-acrylamido-2-methylpropane sulfonic acid) (NIPAMSA) cryogel were used for the reduction of 4-nitrophenol to 4-aminophenol [59]. AgNPs/starch/PEG/PAA hydrogel was created by Saberi et al. for Hg^2+^ removal. The maximum adsorption capacity of Hg^2+^ ions for hydrogel was found to be 158.21 mg/g and 182.53 mg/g in pH 7 and 6 in aqueous solutions, respectively [96]. Similarly, Dil and Sadeghi created nanosilver/gelatin/PAA hydrogel for Cu^2+^ removal. The absorption capacity was 147.10 mg/g in pH 5.5 for 40 min, measured with the atomic absorption spectroscopy technique [97]. As seen, AgNPs-hydrogels, besides their wound applications, are used for metal removal and catalytic activity. Their usage for wound treatment is shown in Table 4.

### 7.1. Chemical Synthesis of AgNPs-Containing Hydrogels

The hydrogel made of polyacrylamide/poly(vinyl alcohol) was used as a template to produce AgNPs through the chemical route. The engineered hydrogel-AgNPs exhibit higher activity on *E. coli* compared to AgNPs alone [98].

Using β-chitin as the starting material, the hydrogel was prepared and loaded with chemically synthesized AgNPs. The prepared β-chitin/nanosilver composite scaffolds exhibited significant bactericidal activity against *E. coli* and *S. aureus* and showed good blood-clotting ability. Cell attachment studies using vero (epithelial) cells showed better adherence of the cells on the scaffolds [99].

### 7.2. Green Synthesis of AgNPs-Containing Hydrogels

A hydrogel made of carbopol was loaded with in-situ prepared AgNPs, using sericin and chitosan as capping and reducing agents, respectively. The antimicrobial activity and wound healing activity of the optimized hydrogel demonstrated higher bactericidal activity and wound closure, as supported by results of histopathology [100].

### 7.3. Irradiation Synthesis of AgNPs-Containing Hydrogels

A novel wound dressing material was prepared using 2-acrylamido-2-methylpropane sulfonic acid sodium salt (AMSAS) hydrogel loaded with AgNPs. The silver nitrate solution was added to the AMSAS solution and the NPs synthesis was initiated through gamma irradiation. This novel silver hydrogel showed excellent antimicrobial activity. A hydrogel made of AMSAS was prepared and loaded with AgNO_3_ solution and the reaction mixture was irradiated with UV radiation for the formation of AgNPs. The antibacterial activity of the hydrogels against common burn pathogens was studied and the results showed that 5 mM AgNPs-loaded hydrogel had the greatest inhibitory activity [101].

The PVA solution mixed with the AgNO_3_ solution was gamma-irradiated for the reduction process of the precursor and then made into hydrogels. The hydrogels were dried in vacuum at 40 °C. PVA-Ag samples were non-toxic and presented antimicrobial activity, confirming that 0.25% AgNO_3_ concentration was enough to establish a potential antimicrobial effect. The samples also presented suitable mechanical and swelling properties in all media, representative for potential burn site applications [102].

### 7.4. Thermal Synthesis of AgNPs-Containing Hydrogels

Gelatin solution was mixed with the AgNO_3_ solution, wherein the precursor was reduced at 40 °C. Furthermore, hydrogels were prepared from the same, which were effective against *S. aureus, E. coli* and *P. aeruginosa*, with at least 99.7% bacterial growth inhibition. The hydrogels containing AgNO_3_ at 0.75 and 1.0 wt% were not detrimental to the skin cells that had been cultured directly on them [103].

The in-situ preparation of AgNPs inside carboxymethylcellulose (CMC) gel was carried out. The antimicrobial activity studies showed that CMC-SNP containing 50 ppm of AgNPs was effective against the growth of both Gram-negative and Gram-positive strains including methicillin-resistant *S. aureus*. These results indicated that the reported nanoparticles could be an ideal option for the treatment of deep infected wounds [104].

In total, 28 publications with mention of the tested microorganisms (Table 5) were searched to determine which bacteria and fungi are used for testing materials for wound dressings (publications from Table 1, Table 2, Table 3, Table 4 were used, except [67,72,78,86,105], where no microorganisms but eukaryotic cells were tested). As seen in Table 5, *S. aureus* is the most favored model as a Gram-positive bacterium because it is a high-priority pathogen [106]. Very favored models for wound testing are also Gram-positive *E. coli* and *P. aeruginosa*. Besides bacteria, fungi models are also tested (*C. albicans*).

## 8. Safety of AgNPs in Wound Dressing Applications

The question of the potential toxicity of AgNPs is widely discussed. Nanomaterials can easily pass through cell membranes and adversely affect human health [107]. In mouse models, they passed through the blood–brain and blood–testis barriers [108]. AgNPs in higher concentrations (>44.0 μg/mL) are necrotic to cells, resulting in cell membrane rupture [107]. After their passing through the blood–testis barrier, AgNPs are deposited in the testes and can have adverse effects on sperm cells [109]. They have also been shown to damage other cells such as the brain [110], liver [111] and stem cells [112]. However, the findings of many authors indicated that the use of AgNPs as a topical antibacterial agent is safe. The AgNPs-loaded hydrogel is considered an excellent wound dressing [113]. If used in reasonable quantities, silver metal and silver dressings have no negative effects on human health [114,115]. For example, Kokura et al. [116] studied the permeability of AgNPs in human skin and their cytotoxicity in human HaCaT (a spontaneously immortalized human keratinocyte line) keratinocytes under ultraviolet B (UVB) irradiation. Based on a transcutaneous passage assay using human skin, AgNPs did not penetrate healthy intact human skin. AgNPs showed no effect on keratinocytes and did not increase UVB-induced cell death. The efficacy of AgNPs increases at low concentrations. AgNPs do not easily penetrate the skin barrier and have no detrimental effects on keratinocytes [116]. Thus, AgNPs possess the outstanding potential for use in wound dressing applications.

In addition, NPs are generally considered a promising replacement for antibiotics due to their bactericidal activity against a large number of pathogens and the non-induction of microbial resistance [117]. AgNPs, in particular, have received a great deal of attention from scientists due to their inhibitory effect on around 650 microbe species and antibiotic-resistant bacteria [118]. AgNPs inhibit bacterial reproduction by denaturing bacterial DNA, leading to the alteration and subsequently the death of the bacterial cell [119]. Moreover, AgNPs can be an appropriate therapeutic agent or prophylactic because they prevent the colonization of the wound by antibiotic-resistant bacteria and other microbes that impede wound healing [120]. Based on using human keratinocytes and dermal fibroblasts, AgNPs significantly reduced levels of inflammatory cytokines and promoted healing [121]. In vitro cell culture tests exhibited no cytotoxicity in the hydrogel containing AgNPs and non-adherent character to dermal fibroblasts [122]. Compared with conventional topical agents, AgNPs can both effectively prevent wound infections and improve the healing process of injured tissues. AgNPs-coated wound dressings possess considerable antibacterial activity and repair tissues faster and more efficiently [123]. Application of silver modified nanoporous silica carriers loaded with sulfadiazine instead of silver sulfadiazine could overcome the poor aqueous solubility of silver sulfadiazine which limits its antibacterial effect [124].

On the contrary, some authors are convinced of the risks of using AgNPs. Some cases where argyria appeared after treatment of burn wounds with dressings containing nanocrystalline silver were reported [125,126]. The use of these dressings caused the deposition of the silver particles into the mid and deep dermis [125]. For silver itself, it was found that this metal had a concentration-dependent cytotoxic effect on human dermal fibroblast cells [127]. However, thanks to the development of nanotechnology, its minimum inhibitory concentration, as well as toxicity to normal human cells, was reduced [128] and a number of brands of wound dressings containing silver have been accepted by the U.S. Food and Drug Administration [100]. On the other hand, Burd et al. [129] demonstrated that some commercial silver-based dressings exhibited cytotoxic effects on cultured keratinocytes and fibroblasts.

## 9. Conclusions

Nanomaterials nowadays represent a very promising way to eliminate bacterial, fungal and viral infections. Besides their antibiotic, antifungal and antiviral effects, they are used as catalysts, bioremediation elements and pseudo-enzymes. This review represents successful results of the nanotechnology field in the very last years. Acute and chronic wounds are problematic to heal and are often underestimated by people. The types of treatment were divided based on article information into four categories: membranes, powdered, nanofibers and hydrogels, prepared by different types of synthesis. It can be concluded that the chemical method of synthesizing NPs can obtain NPs with uniformity and reproducibility of preparation. However, a growing supply of lowering of environment pollution is not in consensus with the use of this type of synthesis. Green synthesis is much more environmentally and user friendly because it uses a natural source of reduction agents. A wide spectrum of different types of NPs can be obtained. It is necessary to mention that optimization of the production of uniform green-synthesized NPs can take a long time because slight, unwitting changes in laboratory protocol can lead to the production of NPs with completely different chemical and physical properties. Furthermore, the detailed characterization of an extract of plant or fungus is needed. Irradiation synthesis of nanoparticles shows an interesting way to produce unique, unrepeatable NPs, which can be considered as the main advantage. The main disadvantages are the complicated legislature connected with work with radiation sources and increase the risk of harm to health. Thermal synthesis is based on artificial or natural chemicals as reducing agents connected with increased temperature. In its essence, it is a combination of green and chemical synthesis methods, where the higher temperature is the main element of reduction power. Potential advantages and disadvantages ensue from other components used in thermal synthesis. Despite a plethora of different publications in recent years, it is still necessary to find new polymers and new types of synthesis of (not only) AgNPs, because of the growing bacterial resistance to metal NPs. It is important mainly not to forget to conduct valid tests on eukaryotic cells to determine how to achieve the best effect against prokaryotic cells and at the same time ensure no harmful effect against eukaryotic cells. Suitable models can be keratinocytes and fibroblasts, which are also mentioned as models in this publication.

## Figures and Tables

**Table 1:** AgNPs-membrane composites for wound treatment.

AgNPs Preparation	AgNPs Size (nm)	Polymer Used	Incorporation Method	Main Result
CHEMICAL METHOD (NaBH_4_)	5–14	BC	BC membrane was mixed with AgNO_3_ and reduced with NaBH_4_.	Inhibition of *S. aureus* and *E. coli* growth on different sugar media (glucose, sucrose, maltose) [66]
CHEMICAL METHOD (NaBH_4_)	15	Chitosan	Chitosan was mixed with AgNPs prepared by reduction of AgNO_3_ with NaBH_4_ and the mixture was left to dry.	AgNPs in chitosan wound dressing materials facilitate cell proliferation and mitigate bacterial infection [67].
CHEMICAL METHOD (sodium citrate)	10–30	PVP chitosan	PVP and chitosan were mixed in a 1:1 ratio, then AgNPs prepared by reduction of AgNO_3_ with sodium citrate were added, and afterward, the mixture was dried for 48 h.	Addition of 0.001 to 0.01 mg AgNPs to PVP-chitosan film significantly reduced the growth of *E. coli and S. aureus* [68].
CHEMICAL METHOD (sodium citrate)	5	Chitin	Chitin/nanosilver composite scaffolds were prepared by addition of the nanosilver solution (prepared by reduction of AgNO_3_ with sodium citrate) to chitin hydrogel to obtain chitin/nanosilver composite scaffolds.	This composite inhibits the growth of *S. aureus* and *E. coli*. Inhibition zone on the plate was higher in *E. coli* than *S. aureus*, indicating higher susceptibility of Gram-negative bacteria to nanosilver [69].
CHEMICAL METHOD (NaBH_4_)	3–17	BC	AgNPs were impregnated into BC fiber by immersing BC pellicles in AgNO_3_ for 1 h. The silver ion-saturated BC pellicles were reduced with NaBH_4_.	The growth inhibition ring of *E. coli* and *S. aureus* was 2 and 3.5 mm, respectively. No inhibition zone was observed with the pure BC as a control [70].
GREEN METHOD (egg white)	8–32	KGM	KGM/AgNPs composite sponge	Animal models showed that the KGM/AgNPs composite sponges effectively accelerated wound healing, fibroblast growth promotion and wound epithelialization on the rabbit model [71].
GREEN METHOD (*Camelia sinensis*)	60–150	Chitosan and chitin	Film casting and dipping in AgNO_3_ solution	Evaluation of nanofilms as a temporary biological wound dressing material for rats had revealed good healing activity [72].
GREEN METHOD (chitosan)	16	Chitosan, PVA, CU	Film casting by evaporation	AgNPs prepared from chitosan demonstrated significant effects against various common pathogens (*E.coli*, *S. aureus*, *P. aeruginosa, C. albicans*) [73].
GREEN METHOD (cellulose from *A. xylinum*)	50–150	BC	AgNO_3_ and AgCl reduced by BC and directly incorporated into BC	Membranes exhibited high hydrophilic ability and strong antimicrobial activity against *S. aureus* and *E. coli* [74].
IRRADIATION METHOD (gamma rays, ^60^Co)	3–13	Chitin	Gamma rays prepared AgNPs were mixed with chitin in 5% LiCl and DMA system.	Bactericidal effect was significant (*p* < 0.01) in the presence of chitin nanosilver dressings, whereas the counts of bacteria progressively increased in the absence of nanosilver dressings [75].
THERMAL METHOD (thermal reduction; 80 °C)	10–30	Purchased BC	Freeze-dried BC membrane impregnated with AgNPs (direct incorporation)	The thermally prepared AgNPs exhibited significant antibacterial activity, with more than 99% reduction in *S. aureus.* Moreover, composites allowed attachment and growth of epidermal cells with no cytotoxicity that emerged [76].

Explanation of abbreviations: BC—bacterial cellulose; CU—curcumine; DMA—dimethylacetamide; KGM—konjac glucomannan; PVA—poly(vinylalcohol); PVP—poly(vinylpyrrolidone).

**Table 2:** Powdered AgNPs for wound treatment application.

Method of AgNPs Preparation	AgNPs Size (nm)	Material Used	Incorporation Method	Main Result
CHEMICAL METHOD (alkali solution of starch)	22–24	cotton fabrics	Pressure incorporation of AgNPs to cotton fabrics	13- and 10-mm inhibition zones for *S. aureus* and *E. coli* were obtained, when cotton fabrics with 250 ppm AgNPs were used [77].
CHEMICAL METHOD (NaBH_4_)	4–24	dressing material	AgNPs-coated dressing material	AgNPs showed antimicrobial properties, reduction in wound inflammation and modulation of fibrogenic cytokines [78].
GREEN METHOD (*Fusarium oxysporum*)	2	cotton fabrics	Incorporation to cotton fabrics	AgNPs-impregnated fabrics showed a 99.9% reduction of *S. aureus* growth [79].
GREEN METHOD (*Aspergillus niger*)	200–800	AgNPs-incorporated wound dressings	AgNPs-coated dressing material	AgNPs synthesized from *Aspergillus niger* possess effective wound healing activity when compared with AgNO_3_ [80].

**Table 3:** AgNPs nanofibers for wound treatment.

Method of AgNPs Preparation	AgNPs Size (nm)	Polymer Used	Incorporation Method	Main Result
CHEMICAL METHOD (sodium citrate)	25–55	Collagen	Electrospun fibers	Histology analysis revealed accelerated wound healing [85].
CHEMICAL METHOD (*N*,*N*-DMF)	3–5	PVP	Ag^+^ in PVP solution were reduced to nanofibers with *N*,*N*-DMF.	AgNPs-PVP showed the ability to be used in antibacterial separation filters [86].
CHEMICAL METHOD (Ag^+^ dipped in PMMDM)	<20	PMMDM	Electrospun fibers were dipped in AgNO_3_ solution and dried in a vacuum.	AgNPs showed high antibacterial activity without significant effects on mammalian cells [87].
CHEMICAL METHOD (PBG reduction)	60	PBG	AgNO_3_ was reduced with PBG and then crosslinked with collagen.	Antibacterial and pro-wound healing activities in the PBG crosslinked collagen scaffold suggest the importance of nanobiotechnology for the development of biomaterials for tissue engineering applications [88].
CHEMICAL METHOD (AgNO_3_ reduced with gelatin powder)	11–20	Gelatin fibers	Electrospun fibers further crosslinked glutaraldehyde.	High antibacterial activity in response to Gram-positive (methicillin- resistant *S. aureus*) and Gram-negative bacteria [89].
CHEMICAL METHOD (AgNO_3_ reduced with PEO and DMF)	13–17	PEOPCL	Electrospun fibers	Composite nanofibers possessed good roughness, wettability and antibacterial potential [90].
CHEMICAL METHOD (NaBH_4_)	5–17	Alginate	AgNO_3_ was loaded on fibers and afterward reduced to Ag^0^ with NaBH_4_.	The alginate fibers loaded with AgNPs reduce the inflammatory phase and increase epidermal thickness, improving the overall quality and speed of healing [91].
GREEN METHOD (chitosan, glucose)	10–30	Chitosan, glucose, PVA	AgNPs were used for electrospinning nanofiber materials.	The antibacterial experiment indicated that the electrospun mats of PVA/chitosan blends had good bactericidal activity against the Gram-negative *E. coli* [92].
GREEN METHOD (PGA, HA)	5–13	PGA	PGA was mixed with AgNO_3_ to obtain AgNPs, then mixed with HA, and the mixture was electrospun to obtain nanofibers.	The in vivo study in albino rats showed maximum wound epithelization and collagen deposition after 14 days of nanofiber administration [93].
GREEN METHOD (*P. nigrum*)	5–20	PCL	AgNPs were mixed with PCL during electrospinning to obtain nanofibers.	The fabricated material showed excellent antibacterial activity against both *S. aureus* and *E. coli*, which suggests the ability of the fabricated material to prevent bacterial colonization in wounds covered with this material [94].
IRRADIATION METHOD (gamma rays, ^60^Co)	23–24	PVA	AgNO_3_, PVA and ethanol were mixed, nitrogen-flushed and radiated by ^60^Co.	The manufacture and evaluation of silk-based wound dressings in this study showed that the incorporation of AgNPs at low concentrations on electrospun SF mats could confer significant antibacterial activity against *S. aureus* and *P. aeruginosa* [95].

Explanation of abbreviations: DMF—dimethylformamide; HA—hyaluronic acid; PBG—plumbagine; PCL—poly(caprolactone); PEO—poly(ethylene oxide); PEOPCL—poly(ethylene oxide)-poly(caprolactone); PGA—polygalacturonic acid; PMMDM—poly(methyl methacrylate-co-dopamine methacrylamide); PVA—poly(vinylalcohol); PVP—poly(vinylpyrrolidone);SF—silk fibroin

**Table 4:** AgNPs hydrogels for wound treatment.

Method of AgNPs Preparation	AgNPs Size (nm)	Polymer Used	Incorporation Method	Summary
CHEMICAL METHOD (NaBH_4_)	2–3	PAA and PVA	Hydrogels were soaked in AgNO_3_ solution to absorb Ag^+^ and afterward reduced with NaBH_4_.	Hydrogel alone exhibited no antibacterial activity; however, Ag^+^-hydrogels and AgNPs-hydrogels showed significant antibacterial activity against *E. coli* [98].
CHEMICAL METHOD (sodium citrate)	4–8	β-chitin	β-chitin/nanosilver composite scaffolds were prepared by adding nanosilver to β-chitin hydrogel and stirring well for 15 min.	The composite scaffold showed inhibitory effects on bacterial growth, signifying its role as an antibacterial agent. Cytotoxicity studies on the vero cell line proved that the composite scaffold was non-toxic [99].
GREEN METHOD (sericin and chitosan)	240–970	Chitosan	The chitosan-sericin solution was mixed with AgNO_3_	Hydrogel (S/C-SNPs G-1) demonstrated bactericidal activity [100].
IRRADIATION METHOD (UV radiation)	Not given	AMSAS	AgNO_3_ was added to AMSAS solution, with MBA as a crosslinker.	The novel silver hydrogel is an effective antimicrobial dressing and these results support the possibility of using the novel silver hydrogel as a burn wound dressing [101].
IRRADIATION METHOD (UV radiation, gamma rays ^60^Co)	90	PVA	The mixed solution of AgNO_3_ and PVA was UV-radiated, dried and gamma rays radiated.	Samples containing AgNPs showed antimicrobial activity against *E. coli*, *S. aureus* and *C. albicans*. No sample was toxic to mouse fibroblasts [102].
THERMAL METHOD (reduction at 40 °C)	5–14	Collagen	AgNO_3_ was mixed with gelatin powder and the mixture was reduced at 40 °C.	Hydrogels inhibited at least 99.8% of the bacterial growth against *E. coli*, *S. aureus* and *P. aeruginosa* [103].
THERMAL METHOD (reduction at 70—100 °C).	7–21	CMC	AgNO_3_ was mixed with PEG and CMC and reduced at 70–100 °C.	The absorbing property of SNP-CMC gel would help in removing the exudates and preventing wound maceration, while the donation property would help in debridement of dead tissue. These properties facilitate early wound healing [104].

Explanation of abbreviations: AMSAS—2-acrylamido-2-methylpropane sulfonic acid sodium salt; CMC—carboxymethylcellulose; MBA—*N*,*N*′-methylenebis(acrylamide); PAA—poly(acrylic acid); PEG—poly(ethylene glycol); PVA—poly(vinylalcohol); SNP—silver nanoparticles.

**Table 5:** Frequency of tested bacteria and fungi for wound treatment.

Cell Species	AA	RA (%)	References
*S. aureus* *	24	85.7	[66,68,69,70,71,73,74,75,76,77,79,80,85,87,89,92,93,94,99,100,101,102,103,104]
*E. coli*	22	78.6	[66,68,69,70,71,73,74,76,77,79,80,87,88,89,90,93,94,98,99,102,103,104]
*Micrococcus*	1	3.6	[73]
*P. aeruginosa*	9	32.1	[73,75,76,80,85,87,89,101,103]
*C. albicans*	4	14.3	[73,77,101,102]
*Enterococcus*	1	3.6	[101]
*B. subtilis*	3	10.7	[80,88,93]
*A. baumannii*	1	3.6	[101]

* including methicillin-resistant *S. aureus*, AA—absolute abundance, RA—relative abundance.

## Abbreviations

AgNPs	silver nanoparticles
AMSAS	2-acrylamido-2-methylpropane sulfonic acid sodium salt
BC	bacterial cellulose
CMC	carboxymethylcellulose
CU	curcumine
DMA	dimethylacetamide
DMF	dimethylformamide
GO	graphene oxide
HA	hyaluronic acid
HaCaT	the immortal human keratinocyte line
IPA	isopropanol
KGM	konjac glucomannan
MBA	*N*,*N*′-methylenebis(acrylamide)
NIPAMSA	poly(*N*-isopropylacryamide-*co*-2-acrylamido-2-methylpropane sulfonic acid)
NPs	nanoparticles
PAA	poly(acrylic acid)
PBG	plumbagine
PCL	poly(caprolactone)
PDMS	poly(dimethylsiloxane)
PEG	poly(ethylene glycol)
PEO	poly(ethylene oxide)
PEOPCL	poly(ethylene oxide)-poly(caprolactone)
PGA	polygalacturonic acid
PMMDM	poly(methyl methacrylate-*co*-dopamine methacrylamide)
PVA	poly(vinylalcohol)
PVP	poly(vinylpyrrolidone)
SF	silk fibroin
